# Frequency and determinants of Hepatitis B and C virus in general population of Farash Town, Islamabad

**DOI:** 10.12669/pjms.316.7047

**Published:** 2015

**Authors:** Munazza Asad, Farah Ahmed, Humaira Zafar, Sabir Farman

**Affiliations:** 1 Dr. Munazza Asad, MBBS, FCPS. Associate Professor of Physiology, Al-Nafees Medical College and Hospital, Isra University, Islamabad, Pakistan; 2 Dr. Farah Ahmed, MBBS, MSPH, MBA. Assistant Professor of Community Medicine, Al-Nafees Medical College and Hospital, Isra University, Islamabad, Pakistan; 3 Dr. Humaira Zafar, MBBS, MPhil. Assistant Professor of Pathology, Al-Nafees Medical College and Hospital, Isra University, Islamabad, Pakistan; 4 Mr. Sabir Farman, Statistician, Al-Nafees Medical College and Hospital, Isra University, Islamabad, Pakistan

**Keywords:** Hepatitis B, Hepatitis C, Risk Factors

## Abstract

**Background and Objective:**

Both Hepatitis B virus (HBV) and hepatitis C virus (HCV) infections are rapidly spreading in the developing countries. Both of them are blood borne and are transmitted through un-screened blood transfusion, inadequately sterilized needles and equipment. According to WHO’s criteria of endemicity, Pakistan has high disease burden of Hepatitis B and C. The present study was planned to determine the frequency and to identify the risk factors of hepatitis B and C virus in the general community of Farash town.

**Methods:**

This descriptive study was carried out in Al Nafees Medical Hospital Lab, from January 2013 to December 2013. Both the genders and all age groups were included in the study. All the patients who fulfilled the inclusion criteria had given a written consent. Data was collected through questionnaire and was analyzed on Statistical Package for Social Sciences (SPSS) version 21.

**Results:**

Three-hundred and forty five patients were studied. Among these 92 (27%) were males and 253(73%) were female, 33% of them had hepatitis C, 9% had hepatitis B. History of injections was reported in all of the patients. Visit to community barbers was present in 58.6% and 41% cases of hepatitis B and C. History of dental procedures was obtained in 7(24%) and 15(13%) patients of hepatitis B and C.

**Conclusion:**

Major contributors for Hepatitis B and C in Farash town are use of unsterilized therapeutic injections and visit to community barbers. Education of the barbers regarding sterilization may help in reducing the burden of infection in this community.

## INTRODUCTION

Hepatitis B virus (HBV) and hepatitis C virus (HCV) Infections are a major health problem and significant cause of morbidity and mortality, especially in developing countries.^1^ Worldwide prevalence of chronic HBV carriers is about 350 million, and about 170 million people are infected with hepatitis C virus which represents 7% and 3% of the total population respectively.^2^ The World Health Organization (WHO) estimates that almost two billion people are infected with hepatitis B and more than 350 million have lifelong chronic liver infection.^3^ Prevalence of hepatitis B is 4 times higher in black as compared to whites (11.9% compare to 2.6%).^4^ Hepatitis B and C are endemic in Pakistan and it is in the intermediate prevalence zone. The overall prevalence in Pakistani population varies between 2.6% and 5.3% for HBsAg and anti-HCV antibodies.^5^ Infections with hepatitis B virus and hepatitis C virus can lead to chronic liver disease (CLD) and hepato-cellular carcinoma (HCC). HCV progresses to Chronic Liver Disease in 50-80% of cases and may end up in cirrhosis.^6^ The estimated risk of HCV in Pakistan is 2.4-6.5%.^7^ Both the viruses are transmitted through blood either by percutaneous or body fluids (semen, saliva or vaginal secretion). Horizontal transmission of hepatitis B virus in early childhood is common in areas endemic for the virus.^8^

In developing countries, the reasons for increased frequency are multifactorial like transfusion of unscreened/improperly screened blood, administering injections through un-sterilised or used syringes by health care workers and quacks, body piercing with unsterilized needles and shaving by barbers, use of unsterilized instruments for minor or major surgeries, use of unsterilized endoscopes, cystoscopes and dialysis.^3^,^4^ Similarly, sharing syringes by intravenous drug abusers is a significant risk factor for Hepatitis B and C globally.^5^ Overuse and unsafe injection practices cause an estimated 8 to 16 million Hepatitis B virus infections, 2 to 5 million Hepatitis C virus infections and 80,000 to 160,000 HIV infections globally.^9^ The purpose of the current study was to evaluate the magnitude of HBV and HCV and to identify the risk factors in general community of Farash town, which is a suburban area attached to Capital of Pakistan, with a mixed population and no health facilities.

## METHODS

This descriptive/cross sectional study was carried out at Al-Nafees Medical College and Hospital (ANMC&H) after approval from Ethical Review Committee of Isra University from January 2013 to December 2013. Sample size of 345 was calculated using WHO software and patients were selected by Non-probability sampling technique.

Patients of either sex, of all ages, both married and un-married, having different socioeconomic status and previously unscreened patients were included in the study. All drug addicts by history and known cases of hepatitis B and C were excluded from the study.

### Collection of blood specimen and preparation

Blood was collected using aseptic technique by an expert phlebotomist. Sera was separated and analyzed on the same date. Screening for Hepatitis B virus surface antigen (HBsAg) and HCV Antibody (Anti-HCV) was done in the Clinical laboratory of ANMC and Hospital by ELISA technique.

### Statistical Analysis

Data was analyzed by using SPSS version 20. Variables were defined qualitatively and frequency distribution was applied. Analysis was carried out using Chi square test. Relative Risks (RR)and 95% confidence intervals (CI) was calculated for each association. P value < 0.05 was considered as statistical significant.

### Ethical Considerations

The Project was approved by the ethical review board of the University. A written consent for screening from each individual and in case of children from their guardian was obtained.

## RESULTS

A total of 345 patients were screened for HBsAg and anti-HCV, results showed that both HBV and HCV reactive cases are more in males as compared to females and HCV reactive cases were 52% and HBV reactive cases in males were 19%, as shown in Table-I.

**Table-I:** Distribution of reactive and non-reactive cases of HBV and HCV.

HBV &HCV (n=345)	Male	Female
	92 (27%)	253(73%)
HBV Reactive	17(19%)	12(5%)
Non-reactive	75(81%)	241(95%)
HCV Reactive	47(52%)	65(27%)
Non-reactive	45(48%)	188(73%)

**Fig.1:**
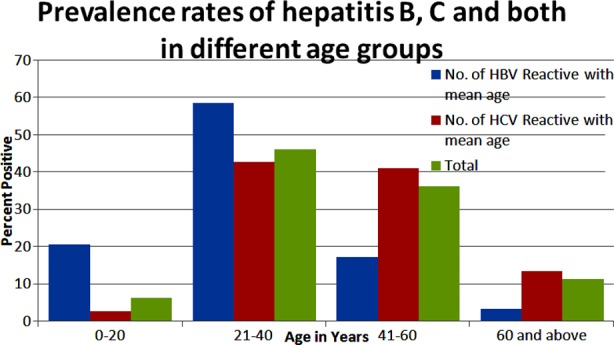
Prevalence rates of Hepatitis B & C and both in different age groups.

Fig.1 results showed that rate for HBV (20%) and HCV (2%) in the 0-20 year age group. In the 21-40 year age group, the infection rates for HBV, HCV were increased to 58% and 42% respectively. The rate for HBV was reduced to 17% in the 41 to 60 year age group, whereas the prevalence of anti-HCV was reduced to 42% only. However, in 60 year and above age group, the rates of both HBV (3%) and HCV (13%) were significantly reduced as compared to 21-40 year age group.

Comparison of various risk factors and their frequency in HBV and HCV infective individuals is shown in Table-II and III. History of injection is present in almost all of the patients having hepatitis B and C. Majority of the patients (58.62% of HBV and 41% of HCV) gave the history of visit to community barbers and 34.48% patients of HBV and 48.21% patients of HCV were reported with history of therapeutic procedures, blood transfusion in only 5% of HCV infected patients, dental procedure history was obtained in 24.13% and 13.39% in HBV and C infections. Of the risk factors measured, In HBV, history of blood transfusion had an RR of 21.79 (CI range 11.29-42.03) and is considered as a significant risk factor (p<0.05). Similarly visit to parlor for various procedures had an RR of 4.35 (CI range 1.45-13.03) which is also significant (p<0.05). For HCV reactive cases, history of therapeutic procedures had an RR of 0.83 (CI range 0.67-1.04). Similarly visit to community barbers in HCV patients had an RR of 2.17 (CI range 1.53-3.07) and is associated with increased risk of infection, p value< 0.05.

**Table-II:** Comparison and Frequency of Risk Factors in HBV (n=29) infective individuals.

Risk factors	HBV	RR	95%CI	P value
History of injections & drips	24 (82.8%)	1.19	0.99-1.43	0.14
History of Therapeutic procedures	10(34.48%)	0.61	0.36-1.02	0.03*
History of dental procedures	07(24.13%)	2.31	1.12-4.75	0.06
History of blood transfusion	20 (69%)	21.79	11.29-42.03	.00*
History of visit to community barbers	17(58.62%)	2.53	1.76-3.65	0.00*
History of line addicts	02(6.89%)	0.94	0.23-3.81	1.00
Positive family history	20 (69%)	54.48	19.96-148.67	.00*
History of visit to parlor	04(13.79%)	4.35	1.45-13.03	0.02*
History of contraceptive injections	15 (51.7%)	14.85	7.53-29.29	0.00*
History of tattooing and body piercing	12(41.37%)	0.53	0.34-0.83	0.00*
History of immunization	01(3.44%)	0.27	0.03-1.91	0.22
History of jaundice and skin disease	01(3.44%)	0.36	0.05-2.56	0.49
History of hospitalization	06 (20.7)	2.51	1.12-5.60	0.04*
History of sharing various objects	02(6.9%)	3.11	0.67-14.30	0.17

• RR Relative Risk: CI Confidence Interval

* • Shows values are statistically significant.

**Table-III:** Comparison and Frequency of Risk Factors in HCV (n=112) infective individuals.

Risk factors	HCV	RR	95% CI	PValue
History of injections	112 (100%)	2.17	1.53-3.07	0.00*
History of Therapeutic procedures	54(48.21%)	0.83	0.67-1.04	0.10
History of dental procedures	15(13.39%)	1.24	0.68-2.27	0.47
History of blood transfusion	05(4.46%)	2.08	0.61-7.03	0.30
History of visit to community barbers	46(41.07%)	2.17	1.53-3.07	0.00*
History of line addicts	08(7.14%)	0.97	0.43-2.20	1.00
Positive family history	01(0.89%)	0.69	0.07-6.59	1.00
History of visit to parlor	04(3.57%)	0.83	0.26-2.59	1.00
History of contraceptive injections	05(4.46%)	1.73	0.54-5.55	0.34
History of tattooing and body piercing	67(59.82%)	0.74	0.62-0.87	0.00*
History of immunization	17(15.17%)	1.47	0.82-2.62	0.21
History of jaundice and skin disease	10(8.92%)	0.99	0.48-2.03	1.00
History of hospitalization	11(9.82%)	1.52	0.72-3.21	0.28
History of sharing various objects	03(2.67%)	1.04	0.26-4.08	1.00

• RR Relative Risk: CI Confidence Interval

* • Shows values are statistically significant.

## DISCUSSION

Hepatitis B virus affects about 350 to 400 million persons worldwide and accounts for about one million deaths from cirrhosis, liver failure, and hepatocellular carcinoma.^10^ Globally the prevalence and number of people with Anti HCV has increased from 2.3 to 2.8%. According to WHO estimates, worldwide there are approximately 54,000 deaths associated with acute HCV infection annually.^11^ Present study revealed that frequency of Hepatitis B virus is 9% and that of hepatitis C infection is 33% in the community of Farash town. Both HBV and HCV infections are rapidly spreading in the developing countries, due to lack of awareness regarding risk factors, health education, poverty, illiteracy and lack of hepatitis B vaccination. Studies showed that in Pakistan the prevalence of Hepatitis B and C virus in general population is around 4% and 5% respectively.^12^

A total of 345 patients were screened for Hepatitis B and C virus infection including 92(27%) males and 243(73%) females. Majority of the screened patients of HBV and C in this study belonged to adult age group (15-49 years). Lack of information regarding health education and unsafe sterilization are among the major contributors in the community.^11^ In our study, the major risk factors identified in this community includes use of unsafe therapeutic injections, improper sterilization methods, in males shaving by barbers, and dental procedures. Most of patients in this study belonged to suburbs areas where above mentioned risk factors do operate and contribute in the spread of Hepatitis B and C. It is a very common practice to give injections even for minor illnesses in these areas of developing countries. Poverty, low education, unsafe health practices, and unscreened transfusions have seriously aggravated the problem.^6^,^13^ Study done by Jafri *et al*. also proved a strong relationship between therapeutic injections and increased frequency of HBV and HCV infections.^14^ In another study Pasha *et al* showed that persons who had more than 4 Injections per year were about 20 times more likely to be infected.^15^ Bari and Shazi *et al*. also showed similar results.^16^,^17^ Luby *et al*. in a follow-up study proved that after community-based efforts to increase awareness, results in decrease use of unsafe injections.^18^

Khan *et al*. also identified injections and dental procedures as a major risk factor for Hepatitis C infections in patients of peri-urban community.^19^ WHO estimates that in South East Asia common person receives 4 injections in one year and majority of them are not adequately sterilized and are not required.^20^

Our study showed that shaving by barbers contributes to 58.6% and 41% in HBV and HCV infections. Lack of immunization against HBV is the main reason for its spread, especially in this high risk community. Study done by Janjua *et al*. showed that only 13% of the barbers knew that hepatitis could be transmitted by contaminated razors and razors were reused for 46% of the shaves.^21^ In Pakistan apart from other known factors shaving by barbers is going to be another major source of spread of this disease.^16^ In China the prevalence of HBsAg, and anti HCV are much higher 16.8% and 39.2% respectively in barbers than in other professions.^22^

In our study, only 5% of the patients develop HCV infection due to blood transfusion and study done by Bosan *et al*. also showed that prevalence of HCV is more as compared to HBV in blood donars.^8^ According to Khatak *et al.*, the prevalence of HBV and HCV in blood donors was 3.3% and 4.0% respectively and Luby *et al*. identified that in Pakistan unscreened blood transfusion is the major cause of HCV infection.^23^

Increased frequency of injection usage has been observed in our study which is found to be one of the major risk factor for HCV spread in this community. Study conducted in Bangladesh showed that the prevalence of HCV infection was significantly higher among the injectable drug users (IDUs) and was associated with sharing of needles and longer duration of injectable drugs used.^24^ Overuse and improperly sterilized injection usage causes an estimated 8-16 million HBV and 2-5 million HCV infections globally which leads to higher burden of morbidity and mortality.^8^ In Australia 80% of Hepatitis B and C individuals are in the population who are parenteral drugs abusers.^25^ Study done by Rathore *et al*. also signified intravenous drug abusers as a major risk factor for the transmission of HCV in the world.^6^

## CONCLUSION

Results of our study showed that past history of therapeutic injections, dental procedures and shaving by barbers are the common risk factors for transmission of HBV and HCV infection in the community. Efforts are needed to educate barbers regarding sterilization of their instruments and resources should be provided for standard screening protocol to reduce the spread of Hepatitis B and C.

## References

[1] Khan MS, Jamil M, Jan S, Zardad S, Sultan S, Sahibzada AS (2007). Prevalence of Hepatitis B and C in Orthopedics patients at Ayub Teaching Hospital Abbotabad. JAMC.

[2] Nafees M, Farooq M, Jafferi G (2009). Frequency of hepatitis B and C infections in the general population of Lahore, Pakistan. Biomedica.

[3] Aziz S, Khanani R, Noorulain W, Rajper J (2010). Frequency of hepatitis B and C in rural and periurban Sindh. J Pak Med Assoc.

[4] Sharif TB, Tariq WUZ (2006). Seroprevalence of Hepatitis B and C in healthy adult male recruits. Pak J Pathol.

[5] Bosan A, Qureshi H, Mohammad K, Ahmad I, Hafiz R (2010). A review of hepatitis viral infections in Pakistan. J Pak Med Assoc.

[6] Rathore JA, Shah MA, Mehraj A (2012). Hepatitis C virus transmission risk factors. JAMC.

[7] Jafri W, Subhan A (2008). Hepatitis C in Pakistan: magnitude, genotype, disease characteristics and therapeutic response. Trop Gastroenterol.

[8] Davis LG, Weber D J, Lemon S M (1989). Horizontal transmission of hepatitis B virus. Lancet.

[9] Kane A, Lloyd J, Zaffran M, Simonsen L, Kane M (1999). Transmission of hepatitis B, hepatitis C and HIV viruses through unsafe injections in the developing world: model based regional estimates. Bull World Health Organ.

[10] Shepard CW, Finelli L, Alter MJ (2005). Global epidemiology of hepatitis C virus infection. Lancet Infec Dis.

[11] Qureshi H, Arif A, Riaz K, Alam SE, Ahmed W, Mujeeb SA (2009). Determination of risk factors for hepatitis B and C in male patients suffering from chronic hepatitis. BMC Res Notes.

[12] Zuberi SJ (1998). An overview of HBV/HCV in Pakistan. Pak J Med Res.

[13] Bostan N, Mahmood T (2010). An overview about hepatitis C: A devastating virus. Crit Rev Microbiol.

[14] Jafri W, Jafri N, Yakoob J, Islam M, Tirmizi SF, Jafar T (2006). Hepatitis B and C: prevalence and risk factors associated with seropositivity among children in Karachi, Pakistan. BMC Infect Dis.

[15] Pasha O, Luby SP, Khan AJ, Shah SA, MC Corbick JB, Fisher-Hoch SP (1999). Household members of hepatitis C virus infected people in Hafizabad, Pakistan: Infection by Injections from healthcare providers. Epidemiol Infect.

[16] Bari A, Akhtar S, Rahbar MH, Luby SP (2001). Risk factors for hepatitis C virus infection in male adults in Rawalpindi-Islamabad, Pakistan. Trop Med Int Health.

[17] Shazi L, Abbas Z (2006). Comparison of risk factors for hepatitis B and C in patients visiting a gastroenterology clinic. J Coll Physicians Surg Pak.

[18] Luby S, Hoodbhoy F, Jan A, Shah A, Hutin Y (2005). long term improvement in unsafe injection practices following community intervention. Int J Infect Dis.

[19] Khan AJ, Luby SP, Fikree F, Karim A, Obaid S, Dellawa S (2000). Unsafe injections and transmission of hepatitis B and C in peri-urban community in Pakistan. Bull WHO.

[20] Ali SA, Donahue RMJ, Qureshi H, Vermund SH (2009). Hepatitis B and C in Pakistan: Prevalence and risk factors. Int J Infect Dis.

[21] Janjua NZ, Nizamy MA (2004). Knowledge and practices of barbers about Hepatitis B and C transmission in Rawalpindi and Islamabad. J Pak Med Assoc.

[22] She SL, Shi LY, Wu YJ, Li ZZ, Zheng CZ, Wu YP (1988). A seroepidemiologic study of hepatitis B virus infection among Barbers in Huangshi City, Heubi China. MicrobiolImmunol.

[23] Khatak MF, Salamat N, Bhatti FA, Qureshi TZ (2002). Seroprevalence of hepatitis B, C and HIV in blood donors in northern Pakistan. J Pak Med Assoc.

[24] Shirin T, Ahmed R, Iqbal A, Islam M, Islam MN (2000). Prevalence and risk factors of hepatitis B virus, hepatitis C virus, and human immunodeficiency virus infections among drug addicts in Bangladesh. J Health PopulNutr.

[25] Dore GJ, Law M, Mac Donald M, Kaldor JM (2003). Epidemiology of Hepatitis C virus infection in Aus. J Clinical Virol.

